# Comparison of the Cumulative Live Birth Rates of Progestin-Primed Ovarian Stimulation and Flexible GnRH Antagonist Protocols in Patients With Low Prognosis

**DOI:** 10.3389/fendo.2021.705264

**Published:** 2021-09-13

**Authors:** Mingze Du, Junwei Zhang, Zhen Li, Xinmi Liu, Jing Li, Wenxia Liu, Yichun Guan

**Affiliations:** The Reproduction Center, The Third Affiliated Hospital of Zhengzhou University, Zhengzhou, China

**Keywords:** progestin-primed ovarian stimulation, GnRH antagonist, low prognosis, cumulative live birth rate, controlled ovarian hyperstimulation

## Abstract

**Objective:**

To compare the cumulative live birth rate (CLBR) of the progestin-primed ovarian stimulation (PPOS) protocol with that of the flexible GnRH antagonist protocol in patients with poor prognosis diagnosed per the POSEIDON criteria.

**Methods:**

This was a retrospective cohort study. Low-prognosis women who underwent IVF/ICSI at the Reproductive Center of Third Affiliated Hospital of Zhengzhou University between January 2016 and January 2019 were included according to the POSEIDON criteria. The CLBR was the primary outcome of interest. The secondary outcome measures were the numbers of oocytes retrieved, 2PN embryos, available embryos and time to live birth.

**Results:**

A total of 1329 women met the POSEIDON criteria for analysis. For POSEIDON group 1, the dosage of gonadotropin (Gn) was higher in the PPOS group than in the GnRH antagonist group (2757.3 ± 863.1 *vs* 2419.2 ± 853.1, P=0.01). The CLBR of the PPOS protocols was 54.4%, which was similar to the rate of 53.8% in the GnRH antagonist group. For POSEIDON group 2, the number of available embryos was higher in the PPOS group (2.0 ± 1.7 *vs* 1.6 ± 1.4, P=0.02) than in the GnRH antagonist group. However, the CLBRs of the two groups were similar (18.1% *vs* 24.3%, P=0.09). For POSEIDON groups 3 and 4, there were no statistically significant differences in the number of oocytes retrieved, 2PN, available embryos or CLBR between the two protocols. After adjustments for confounding factors, the CLBR remained consistent with the unadjusted rates. In the POSEIDON group 1 population, the GnRH antagonist protocols had a shorter time to live birth (P=0.04).

**Conclusion:**

For low-prognosis patients diagnosed per the POSEIDON criteria, the CLBR of PPOS protocols is comparable to that of GnRH antagonist protocols. In the POSEIDON group 1 population, the GnRH antagonist protocols resulted in a shorter time to live birth.

## Introduction

Controlled ovarian stimulation (COS) is a key step in assisted reproductive technology (ART). In routine clinical practice with COS-assisted pregnancy, up to 9%~24% of patients have poor ovarian response (POR) (1). Diagnosis, treatment and fertility assistance for these patients have always been the focus of the field of reproduction, but these issues continue to be challenging. At present, the Bologna criteria, discussed and formulated by the European Society of Human Embryology and Reproduction and the American Society of Reproductive Medicine in 2011, are the most widely used standards in clinical practice (2). This standard facilitates predictions of and consultations regarding clinical outcomes, but it still has certain limitations. Specifically, questions regarding the heterogeneity of these criteria to select homogenous populations for clinical trials have been raised (3–5). Due to the multiple ways in which the Bologna criteria can be met, the baseline characteristics and prognoses of patients are quite different. This heterogeneity is related to differences in underlying causes and may lead to differences in intervention effects. Therefore, the Bologna criteria definition may affect the clinical diagnosis and treatment of specific subpopulations. The POSEIDON criteria were proposed by the POSEIDON group in 2016; they consider ovarian biomarkers, the number of oocytes obtained, the age-related embryo aneuploidy rate and ovarian sensitivity to exogenous gonadotropin (Gn) (6). ‘Low-prognosis women’ are categorized into four groups based on female age, ovarian reserve indicators and the ovarian response to Gn during previous COS. Therefore, the POSEIDON criteria can improve the homogeneity and comparability of clinical research studies, decrease the dilution of potential treatment effects, and provide a more significant guide for formulating clinical protocols for low-prognosis women (6, 7). The development of individualized COS protocols according to different populations is critical to the outcome of assisted pregnancy, especially for patients with low prognosis. Therefore, it is necessary to explore suitable ovulation induction programs for people with low prognosis.

Gonadotropin-releasing hormone (GnRH) antagonist is the routine ovulation stimulation protocol (8, 9). There are a large number of randomized controlled, prospective and retrospective studies on GnRH antagonist regimens that fully prove the effectiveness and safety of GnRH antagonist regimens (10–13). However, it has been reported that GnRH antagonist protocols have a 0.34% to 8.0% chance of failing to control the LH surge, and increased age and diminished ovarian reserve are the main risk factors (14–17). In 2015, Kuang et al. (18) proposed a new COS protocol named progestin-primed ovarian stimulation (PPOS), which has advantages in terms of its effectiveness for suppressing the LH surge as well as its oral administration. Additionally, clinical effectiveness and safety have been demonstrated in women with polycystic ovary syndrome (PCOS), normal ovarian response and POR (19–22). To our knowledge, there have been no studies comparing PPOS and GnRH antagonist protocols according to the POSEIDON criteria, which would be meaningful for the formulation of individualized clinical programs. Therefore, the purpose of this study was to explore the cumulative live birth rate (CLBR) of patients with low prognosis diagnosed per the POSEIDON criteria by comparing patients receiving PPOS protocols with those receiving GnRH antagonist protocols.

## Materials and Methods

### Study Design and Population

This was a retrospective cohort study approved by the review board of the Third Affiliated Hospital of Zhengzhou University. For this study, we included women with a low prognosis who underwent IVF/ICSI at the Reproductive Center of the Third Affiliated Hospital of Zhengzhou University between January 2016 and January 2019 according to the POSEIDON criteria. All women were categorized into four groups according to the POSEIDON criteria (6).

➤POSEIDON group 1: Female age <35 years; ovarian biomarkers showing antral follicle count (AFC)≥5 and/or AMH≥1.2 ng/ml; ovarian response measured as oocytes retrieved < 10.
➤POSEIDON group 2: Female age ≥35 years; ovarian biomarkers showing AFC≥5 and/or AMH≥1.2 ng/ml; ovarian response measured as oocytes retrieved < 10.
➤POSEIDON group 3: Female age <35 years; ovarian biomarkers showing AFC<5 and/or AMH<1.2 ng/ml.
➤POSEIDON group 4: Female age ≥35 years; ovarian biomarkers showing AFC<5 and/or AMH<1.2 ng/ml.

Cycles with adenomyosis, uterine malformations, endometrial polyps, preimplantation genetic testing (PGT) and donor oocytes were excluded. To reduce the confounding impact caused by multiple enrollments of patients, patients were enrolled only once.

### Controlled Ovarian Hyperstimulation Protocols

#### Previous COS Protocols for the POSEIDON Groups 1 and 2

For the POSEIDON groups 1 and 2, the previous COS protocol was the routine GnRH agonist (GnRH-a) protocol, which was divided into the early follicular phase GnRH-a protocol and the mid-luteal phase GnRH-a protocol. The specific protocols have been carefully described in our previous research (23, 24).

#### Progestin-Primed Ovarian Stimulation (PPOS)

For the PPOS protocol, COS was initiated on the second or third day of the menstrual cycle. Patients were administered 6 mg of medroxyprogesterone acetate (MPA) (Beijing Zhong Xin Pharmaceutical, China) combined with human menopausal gonadotropin (hMG) (Anhui Fengyuan Pharmaceutical, China) at a dose of 150 to 300 IU/day depending on maternal age, body mass index (BMI), AMH and basal AFC. Then, follicle growth was monitored by vaginal ultrasound combined with serum hormone analysis 4 days later. If necessary, the dose of hMG was adjusted according to follicle development. When the diameter of the dominant follicle was greater than 20 mm or when at least three follicles reached 18 mm, the final stage of trigger ovulation was performed with triptorelin (100 μg) (Ferring International Center SA, Germany) and 2000 IU of human chorionic gonadotropin (hCG) (Lizhu Pharmaceutical Trading, China), followed by oocyte pickup 36 hours later.

#### GnRH Antagonist

In the flexible GnRH antagonist group, hMG (Anhui Fengyuan Pharmaceutical, China) (150 to 300 IU/day) was initiated on the second or third day of the menstrual cycle. Injection of GnRH antagonist at 0.25 mg/day commenced once the diameter of the dominant follicle reached 14 mm and was continued up to the trigger day. The dose of hMG was adjusted according to the follicle response. As soon as the diameter of the dominant follicle was greater than 20 mm or when at least three follicles reached 18 mm, ovulation induction was co-triggered with triptorelin (100 μg) (Ferring International Center SA, Germany) and 2000 IU hCG (Lizhu Pharmaceutical Trading, China). Oocyte retrieval was performed 36 hours later. Conventional IVF or ICSI was performed based on the sperm quality.

### Embryo Transfer and Endometrial Preparation Protocols

For PPOS protocols, whole embryos were frozen. For the flexible GnRH antagonist group, three days after oocyte retrieval, one or two embryos were transferred under monitoring by abdominal ultrasound or single blastocyst transfer was carried out five days after oocyte retrieval. For cases with severe ovarian hyperstimulation syndrome (OHSS), an endometrial thickness ≤7 mm, progesterone levels ≥2 ng/ml on the hCG trigger day and the presence of uterine fluid, we canceled the fresh embryo transfer, cryopreserved all embryos and subsequently performed frozen embryo transfer (FET). Endometrial preparation for FET was performed by means of the natural cycle for women with regular menstrual cycles and spontaneous ovulation; artificial/induced ovulation cycle for women with irregular menstrual cycles; and downregulation + an artificial cycle for women with endometriosis. Follicle and endometrial scanning was performed by vaginal ultrasound, and embryo or blastocyst transfer was performed using abdominal ultrasound after 3 or 5 days of endometrial development with luteosterone. Routine corpus luteum support, namely, oral dydrogesterone (2 times daily, 10 mg once) (Abbott Co. America) and intravaginal administration of 90 mg of a progesterone sustained-release vaginal gel (Merck Co. Germany), was given. Corpus luteum support was continued at least until 55 days after transfer if pregnancy occurred.

### Outcome Measures and Definition

The primary outcome measure was CLBR, defined as at least one live birth resulting from one aspirated ART cycle, including all cycles in which fresh and/or frozen embryos were transferred, until one delivery with a live birth occurred or until all embryos were used, whichever occurred first. The delivery of a singleton, twin, or other multiple was registered as one delivery (25). We used a conservative approach to assume the CLBR, which means that couples who discontinued treatment would have zero change in conceiving. The observation and follow-up time was 2 years.

The secondary outcome measures were the number of oocytes retrieved, number of 2PN embryos and number of available embryos. The time to live birth is also an observation indicator of this study and is defined as the time from the start of COS to live birth in this cycle (months).

### Statistical Analysis

All statistical management and analyses were performed using SPSS software, version 22.0.

The one-sample K-S test was used to check for normality. Continuous variables with abnormal distributions are expressed as the mean ± SD, and the Student’s t-test was used to assess between-group differences. Categorical variables are represented as the number of cases (n) and percentage (%). The means from chi-square analyses were used to assess the differences between groups with Fisher’s exact test when necessary. For CLBR, binary logistic regression was used to adjust for the baseline characteristics. Adjusted odds ratios (AORs) with 95% confidence intervals (CIs) were calculated. P<0.05 was considered to be statistically significant.

## Results

### Study Population

**Figure 1:**
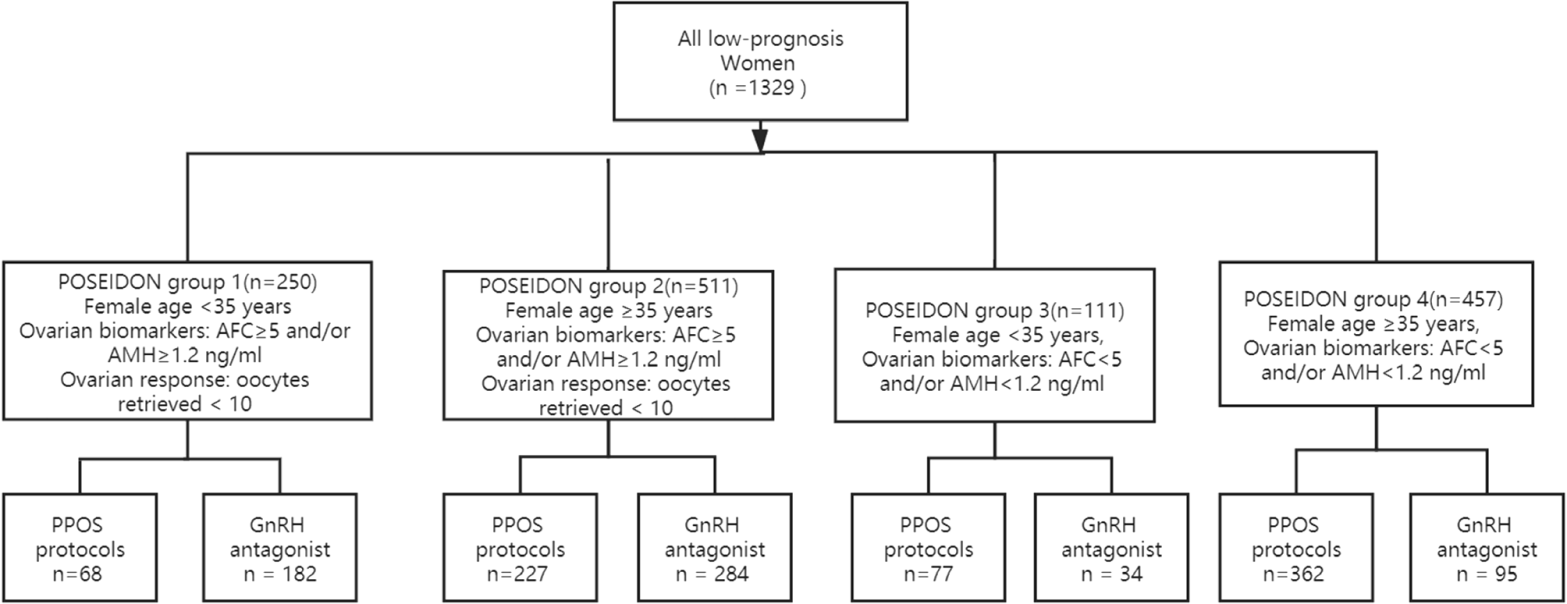
Study flow chart.

In total, 1329 women met the POSEIDON criteria from January 2016 to January 2019 in our reproductive center; 734 women underwent PPOS protocols and 595 women underwent GnRH antagonist protocols. Two hundred fifty women met the criteria for POSEIDON 1 group, 511 women for POSEIDON 2 group, 111 women for POSEIDON 3 group, and 457 women for POSEIDON 4 group. The flowchart of the participants is presented in **Figure 1**.

### Baseline Characteristics

**Table 1** shows the baseline and cycle characteristics of low-prognosis women stratified according to the POSEIDON criteria. Briefly, maternal age and paternal age were higher in POSEIDON groups 2 and 4 than in POSEIDON groups 1 and 3. The AMH level and AFC were higher in groups 1 and 2 than in groups 3 and 4. A total of 55.2% (734/1329) of the women underwent PPOS protocols and 44.8% (595/1329) underwent GnRH antagonist protocols. The baseline and cycle characteristics of the POSEIDON groups 1 and 2 from previous cycles are described in **Supplemental Table 1**.

**Table 1:** Baseline and cycle characteristics of low-prognosis women stratified according to the POSEIDON criteria.

	All low-prognosis Women (n = 1329)	POSEIDON 1 (n = 250)	POSEIDON 2 (n = 511)	POSEIDON 3 (n = 111)	POSEIDON 4 (n = 457)
Maternal age (year)	38.1 ± 5.9	30.0 ± 2.9	40.4 ± 3.4	30.4 ± 3.0	41.8 ± 3.7
Paternal age (year)	39.0 ± 6.6	31.2 ± 4.0	41.3 ± 4.9	31.8 ± 4.4	42.1 ± 5.0
Body mass index (kg/m2)	23.9 ± 3.0	23.3 ± 3.1	24.0 ± 2.9	23.4 ± 3.2	24.2 ± 2.9
Duration of Infertility (year)	4.6 ± 3.2	3.7 ± 2.7	4.8 ± 2.5	4.0 ± 3.1	5.0 ± 3.9
Type of infertility					
Primary infertility	320 (24.1)	133 (53.2)	59 (11.5)	60 (54.1)	68 (14.9)
Secondary infertility	1009 (75.9)	117 (46.8)	452 (88.5)	51 (45.9)	389 (85.1)
Indication of IVF/ICSI					
low prognosis	253 (19.0)	19 (7.6)	60 (11.7)	30 (27.0)	144 (31.5)
low prognosis + tubal factor	441 (33.2)	112 (44.8)	217 (42.5)	34 (30.6)	78 (17.1)
low prognosis + male factor	121 (9.1)	21 (8.4)	73 (14.3)	6 (5.4)	21 (4.6)
low prognosis + others	514 (38.7)	98 (39.2)	161 (31.5)	41 (36.9)	214 (46.8)
Basal serum FSH level (IU/L)	10.1 ± 5.6	6.5 ± 4.3	8.9 ± 4.3	11.8 ± 5.4	12.0 ± 6.3
AMH (ng/ml)	1.7 ± 1.2	3.3 ± 2.6	1.8 ± 1.4	0.8 ± 0.2	0.7 ± 0.2
Basal antral follicle count	6.3 ± 3.6	10.4 ± 6.4	8.1 ± 3.1	3.0 ± 0.9	3.0 ± 1.0
Fertilization method					
IVF	921 (0.7)	185 (0.7)	348 (0.7)	68 (0.6)	320 (0.7)
ICSI	408 (0.3)	65 (0.3)	163 (0.3)	43 (0.4)	137 (0.3)
COS protocols					
PPOS	734 (55.2)	68 (27.2)	227 (44.4)	77 (69.4)	362 (79.2)
GnRH antagonist	595 (44.8)	182 (72.8)	284 (55.6)	34 (30.6)	95 (20.8)

Data are presented as the mean ± SD for continuous variables and n (%) for categorical variables. COS, controlled ovarian stimulation; PPOS, progestin-primed ovarian stimulation.

### Reproductive Outcomes

The detailed reproductive outcomes are shown in **Table 2**. For POSEIDON group 1, the Gn dosage was higher in the PPOS protocols than in the GnRH antagonist group (2757.3 ± 863.1 *vs* 2419.2 ± 853.1, P=0.01). The serum hormone levels (including LH, E2 and P) on the trigger day, number of oocytes retrieved, number of 2PN embryos and number of available embryos were comparable between the two groups. The CLBR in the PPOS group was 54.4%, similar to the 53.8% observed in the GnRH antagonist group. For POSEIDON group 2, the number of available embryos was higher in the PPOS group (2.0 ± 1.7 *vs* 1.6 ± 1.4, P=0.02) than in the GnRH antagonist group. However, the CLBRs were similar between the two groups (18.1% *vs* 24.3%, P=0.09). For POSEIDON groups 3 and 4, there were no statistically significant differences in Gn dosage; duration of ovarian stimulation; LH, E_2_, and P values on the trigger day; number of oocytes retrieved; number of 2PN embryos; number of available embryos; number of embryo transfer cycles that reached live birth or the end of observation or CLBR between the PPOS and GnRH antagonist groups. In POSEIDON group 1, the time to live birth of the GnRH antagonist protocol was significantly shorter than that of the PPOS protocol (P=0.04). In the other three groups, although the time for the GnRH antagonist to reach live birth was shorter than that of the PPOS protocols, the difference was not statistically significant.

**Table 2:** Clinical and pregnancy outcomes between PPOS and GnRH antagonist protocols of low-prognosis women.

	POSEIDON 1			POSEIDON 2			POSEIDON 3			POSEIDON 4		
	PPOS (n = 68)	GnRH antagonist (n = 182)	P value	PPOS (n = 227)	GnRH antagonist (n = 284)	P value	PPOS (n = 77)	GnRH antagonist (n = 34)	P value	PPOS (n = 362)	GnRH antagonist (n = 95)	P value
Dosage of gonadotropins (IU)	2757.3 ± 863.1	2419.2 ± 853.1	0.01	2728.6 ± 850.4	2808.6 ± 804.0	0.28	2773.1 ± 998.5	2644.1 ± 1175.9	0.55	2635.4 ± 959.3	2515.9 ± 1007.6	0.29
Duration of ovarian stimulation (days)	10.0 ± 2.4	10.2 ± 2.5	0.6	9.5 ± 2.6	9.7 ± 2.2	0.46	10.1 ± 3.0	9.9 ± 3.3	0.66	9.5 ± 2.9	9.2 ± 3.0	0.30
LH values on the trigger day (mIU/ml)	2.4 ± 1.5	2.7 ± 1.2	0.33	3.0 ± 2.6	3.4 ± 2.1	0.27	3.5 ± 2.4	5.1 ± 4.7	0.08	4.7 ± 3.8	4.8 ± 4.4	0.85
E2 values on the trigger day (mIU/ml)	2265.2 ± 1170.5	2117. ± 1045.9	0.42	2454.2 ± 1551.2	2581.1 ± 971.5	0.53	2010.0 ± 1209.1	1937.2 ± 1019.9	0.85	1884.5 ± 1002.8	1900.5 ± 1003.6	0.94
P values on the trigger day (ng/ml)	0.9 ± 0.7	1.4 ± 1.2	0.13	1.2 ± 1.0	1.3 ± 1.2	0.54	1.4 ± 1.0	1.5 ± 1.1	0.21	1.5 ± 1.0	1.2 ± 1.1	0.35
No. of oocytes retrieved	4.7 ± 2.7	4.0 ± 2.5	0.07	4.2 ± 2.4	3.7 ± 2.4	0.07	2.8 ± 2.0	2.8 ± 2.1	0.85	1.6 ± 1.4	1.6 ± 1.3	0.88
No. of 2PN	2.6 ± 1.9	2.5 ± 2.0	0.93	2.4 ± 1.9	2.2 ± 1.8	0.25	1.5 ± 1.3	1.5 ± 1.4	0.89	1.4 ± 1.3	1.4 ± 1.2	0.57
No. of available embryos	2.0 ± 1.6	1.7 ± 1.2	0.30	2.0 ± 1.7	1.6 ± 1.4	0.07	1.2 ± 1.1	0.9 ± 0.8	0.07	1.1 ± 0.8	0.9 ± 0.6	0.41
Number of embryo transfers cycles that reached live birth or the end of observation	0.8 ± 0.5	0.7 ± 0.5	0.39	0.9 ± 0.6	0.7 ± 0.6	0.06	0.5 ± 0.1	0.5 ± 0.1	0.11	0.6 ± 0.1	0.5 ± 0.1	0.06
Time to live birth (month)	13.1 ± 4.4	10.4 ± 3.3	0.04	11.1 ± 3.2	10.4 ± 3.6	0.46	12.9 ± 4.5	11.3 ± 3.6	0.47	12.1 ± 5.0	9.5 ± 1.0	0.32
Cumulative live birth rate	37 (54.4)	98 (53.8)	0.94	41 (18.1)	69 (24.3)	0.09	13 (16.9)	8 (23.5)	0.41	38 (10.5)	7 (7.4)	0.36

Data are presented as the mean ± SD for continuous variables and n (%) for categorical variables. The Student’s t-test was used for continuous variables, and the Pearson χ^2^ test was used for categorical variables with Fisher’s exact test when necessary.

Regarding the main outcome measures, namely, the CLBR, we conducted a binary logistic regression analysis with adjustments for confounding factors. These factors included maternal age, BMI, duration of infertility, type of infertility (primary/secondary), infertility diagnosis (low prognosis/low prognosis+tubal factor/low prognosis+male factor/low prognosis+others) and AFC. The AOR values with their 95% CIs are presented in **Table 3**. After adjustments for confounding factors, the CLBR remained consistent with the unadjusted rates, and the rates were comparable between the PPOS and GnRH antagonist groups of patients with low prognosis. For POSEIDON groups 2 and 4, maternal age was associated with the CLBR.

**Table 3:** Binary logistic regression analysis to account for confounding variables affecting the cumulative live birth rate.

	POSEIDON 1		POSEIDON 2		POSEIDON 3		POSEIDON 4	
	AOR	95% CI	AOR	95% CI	AOR	95% CI	AOR	95% CI
Maternal age (year)	1.01	0.92-1.11	0.89	0.83-0.95	1.02	0.86-1.20	0.81	0.74-0.90
Body mass index (kg/m^2^)	1.05	0.96-1.14	0.95	0.88-1.03	1.02	0.87-1.20	0.96	0.86-1.08
Type of infertility (primary/secondary)	0.76	0.46-1.28	0.87	0.46-1.66	0.99	0.35-2.78	1.40	0.56-3.49
Infertility diagnosis (low prognosis/low prognosis +tubal factor/low prognosis+ male factor/low prognosis+ others)	1.03	0.86-1.24	1.05	0.89-1.24	0.80	0.53-1.21	1.11	0.82-1.51
AFC	1.00	0.96-1.05	1.02	0.95-1.10	1.45	0.81-2.58	1.02	0.86-2.18
COS protocols (PPOS/GnRH antagonist)	1.02	0.56-1.85	1.27	0.81-2.00	1.38	0.49-3.89	0.47	0.20-1.12

Variables entered in the logistics regression model are listed. AOR, adjusted odds ratio; CI, confidence interval.

## Discussion

In summary, the CLBRs of the PPOS and GnRH antagonist groups were comparable among women with low prognosis per the POSEIDON criteria. For POSEIDON group 2, the number of available embryos was higher in the PPOS group. At the same time, we also analyzed the time to live birth for the two COS protocols. Since GnRH antagonists have the opportunity for fresh embryo transfer, overall, the GnRH antagonist regimen has a shorter time to live birth than the PPOS protocols, but there was a significant difference between the two groups in only POSEIDON 1. In the other three groups, there was no significant difference in the time to live birth between the two COS protocols. This also requires further clinical studies with large samples.

### Comparisons With Other Reports

To our knowledge, regarding the comparison of PPOS protocols and GnRH antagonist protocols, there are 11 studies, including four RCTs (20, 22, 26, 27), two prospective studies (28, 29) and five retrospective cohort studies (19, 21, 30–32). The populations in these studies had different characteristics, including PCOS, POR, normal ovarian response and donor oocyte cycles. The progestins used in the studies were mainly MPA and dydrogesterone, and one study used desogestrel. A recent meta-analysis showed that the duration of stimulation, Gn consumption and oocyte yield were similar to those of the PPOS and GnRH antagonist protocols (33). In our study, for POSEIDON group 2, the number of available embryos was higher in the PPOS group; in the other groups, the number of available embryos was not significantly different. A total of five studies, including 1016 women, explored the clinical pregnancy rate, which was similar to the rates in the PPOS and GnRH antagonist groups (RR: 1.12, 95% CI: 0.91 to 1.38) (19, 21, 26–28, 33). The live birth rate (LBR) per embryo transfer cycle (RR: 1.36, 95% CI: 0.88 to 2.11) was similar to those of the PPOS and GnRH antagonist protocols (19, 26, 33). Only one study, which included 318 young women with regular menstrual cycles, explored the CLBR between the PPOS and GnRH antagonist protocols. The CLBRs were 70.6% and 68.7%, respectively, and there was no significant difference. To our knowledge, only one study explored PPOS and GnRH antagonist protocols in women with POR, and that study included 340 poor responders who met the Bologna criteria and were randomly divided into two groups for analysis. The LBRs of the PPOS and GnRH antagonist groups were similar (21.8% *vs*. 18.2%, RR: 1.25, 95% CI: 0.73-2.13, P>0.05) (26). To our knowledge, the POSEIDON criteria are more meaningful for the formulation of individualized clinical protocols. Therefore, this study examined the population according to the POSEIDON criteria, and the classification was more specific and detailed, which can provide more reference for clinical decision-making and treatment. In our study, the main concern was the CLBR, which contributes to the comprehensive evaluation of ovulation induction methods and laboratory techniques to provide better data support for the formulation of treatment strategies. In our study, the CLBRs of the two protocols for patients with low prognosis diagnosed per the POSEIDON criteria were comparable. Overall, the GnRH antagonist regimen had a shorter time to live birth than the PPOS protocols, though there was only a significant difference between the two groups at Poseidon 1.

### Strengths and Limitations

To our knowledge, this is the first study to compare the CLBRs of PPOS and GnRH protocols in patients with low prognosis per the POSEIDON criteria. Compared with the Bologna criteria formulated in 2011, the POSEIDON criteria proposed in 2016 provide a stronger basis for the formulation of clinical protocols and individualized fertility treatments. Moreover, this study used the CLBR as the final observation index to more completely evaluate the entire ovulation induction cycle. However, this study also has certain limitations. First, this study was a retrospective cohort study, and there was interference from confounding factors. To reduce the influence of important confounding factors, this study used logistic regression analysis for adjustments. Second, due to the influence of the PPOS protocols on endometrial receptivity in the uterus, it is necessary to freeze all embryos and perform freeze-thaw embryo transfer in the subsequent cycle. Since the electronic medical record system of this reproductive center cannot display a patient’s specific expenses, this study did not analyze the specific medical expenses.

### Future Prospects

Regarding future research directions, the following points are worth considering. First, a larger sample size or a large, well-designed multicenter RCT study is needed. The second point concerns the economic potency ratio, which is also an important aspect that needs to be considered in the choice of the plan because MPA drugs in the PPOS protocol are more convenient and cheaper to take orally. However, due to the impact of MPA on the receptivity of the endometrium, fresh cycle transfer cannot be performed. The resulting embryo freezing costs, preservation costs, and round-trip expenses need to be further evaluated and analyzed in future research. The third and most important aspect is the offspring safety of the two protocols. Since the birth of ART technology, offspring and perinatal safety have been the focus and difficult to research. Treatment with a GnRH antagonist is a routine ovulation stimulation protocol and has been used to prevent premature LH surge since the 1990s (8, 9). There are a large number of randomized controlled, prospective and retrospective studies on GnRH antagonist regimens that fully prove the effectiveness and safety of these regimens (10–13). Regarding the PPOS protocols, although it has not been applied for a long time, studies have shown that it can achieve exact effectiveness and safety in different populations (19–22). However, due to the short application time of the protocols and the limited amount of data, the long-term safety of offspring deserves further study.

## Conclusion

In conclusion, for low prognosis patients diagnosed per the POSEIDON criteria, the CLBRs of the PPOS and GnRH antagonist protocols were comparable. For POSEIDON group 2, the number of available embryos was higher in the PPOS group. In the POSEIDON group 1 population, the GnRH antagonist protocols had a shorter time to live birth. Comparison of these two effective COS protocols requires further randomized controlled studies with large samples.

## Data Availability Statement

The original contributions presented in the study are included in the article/**Supplementary Material**. Further inquiries can be directed to the corresponding author.

## Ethics Statement

The studies involving human participants were reviewed and approved by Ethics committee of The Third Affiliated Hospital of Zhengzhou University. Written informed consent for participation was not required for this study in accordance with the national legislation and the institutional requirements.

## Author Contributions

MD and YG designed the study and selected the population to be included and excluded. JZ and ZL performed the data extraction and analysis. EL, JL, and XL reviewed the data. MD and JZ drafted this article. All authors contributed to the article and approved the submitted version.

## Conflict of Interest

The authors declare that the research was conducted in the absence of any commercial or financial relationships that could be construed as a potential conflict of interest.

## Publisher’s Note

All claims expressed in this article are solely those of the authors and do not necessarily represent those of their affiliated organizations, or those of the publisher, the editors and the reviewers. Any product that may be evaluated in this article, or claim that may be made by its manufacturer, is not guaranteed or endorsed by the publisher.

## Supplementary Material

Supplementary Table 1 Baseline and cycle characteristics of the Poseidon 1 and 2 groups from previous cycles.
