# Supplementary material for: Comparison of the Cumulative Live Birth Rates of Progestin-Primed Ovarian Stimulation and Flexible GnRH Antagonist Protocols in Patients With Low Prognosis
Source: Front Endocrinol (Lausanne). 2021 Sep 13;12:705264. doi: 10.3389/fendo.2021.705264 (PMC8475782; doi:10.3389/fendo.2021.705264)
Supplement: Supplementary Table 1 — Baseline and cycle characteristics of the Poseidon 1 and 2 groups from previous cycles. [file Table_1.docx]

| Supplementary Table1 Baseline and cycle characteristics of the Poseidon 1 and 2 groups from previous cycles | | |
| --- | --- | --- |
|  | POSEIDON 1 (n =250) | POSEIDON 2 (n =511) |
| Maternal age on oocytes retrieval day (year) | 29.8±2.8 | 40.3±3.1 |
| Body mass index (kg/m^2^) | 23.4±3.0 | 24.1±2.8 |
| COS protocols |  |  |
| Early follicular phase GnRH-a | 172(68.8) | 342(66.9) |
| Mid-luteal phase GnRH-a | 78(31.2) | 169(33.1) |
| Dosage of gonadotropins (IU) | 2924.5±789.5 | 3298.8±932.5 |
| Duration of ovarian stimulation (days) | 13.8±2.2 | 13.3±2.0 |
| No. of oocytes retrieved | 4.2±1.5 | 3.7±2.3 |
| No. of 2PN | 2.8±1.7 | 2.5±1.8 |
| No. of available embryos | 1.8±1.0 | 1.7±1.2 |
